# Coupling Between Thickness-Shear and Flexural Modes in AT-Cut Quartz Mesa Resonators with Beveled Edges

**DOI:** 10.3390/mi17091090

**Published:** 2026-09-16

**Authors:** Xin Fu, Hang Chen, Wanli Yang, Chao Zhan, Xiaowei Zhang, Xuan Mao, Hongping Hu

**Affiliations:** 1 Department of Engineering Mechanics, School of Aerospace Engineering, Huazhong University of Science and Technology, Wuhan 430074, China; fux@hust.edu.cn (X.F.); m202472033@hust.edu.cn (H.C.); wanli_yang_nt1@163.com (W.Y.); 2 Hubei Key Laboratory of Engineering Structural Analysis and Safety Assessment, Huazhong University of Science and Technology, Wuhan 430074, China; 3 TKD Science and Technology Co., Ltd., Suizhou 441300, China; zc@sztkd.com (C.Z.); zxw@sztkd.com (X.Z.); mx@sztkd.com (X.M.); 4 Hubei Key Laboratory of Micro-Nano Crystal Processing Technology, Suizhou 441300, China

**Keywords:** AT-cut quartz resonator, mesa structure, beveled edge, thickness-shear mode, mode coupling

## Abstract

Beveled edges are inevitably formed in micro AT-cut quartz mesa resonators (QMRs) during the photolithography process. This local geometric variation alters the thickness distribution and edge stiffness, which in turn affects the coupling between the primary thickness-shear mode and adjacent parasitic modes. To elucidate the mechanism, we establish a vibration analysis model for the micro AT-cut QMR with beveled edge profiles based on the first-order Mindlin plate theory. The coupling between thickness-shear and flexure is the focus of the investigation. The uniform thickness regions and the beveled edge region are expressed using analytical solutions and power series expansions, respectively. The eigenfrequency equation is then derived through the interface continuity and free boundary conditions. The theoretical frequency spectra show good agreement with finite element results so that the accuracy of the proposed analytical approach is validated. A mode coupling intensity index is constructed based on the discrete Fourier spectrum of the surface displacement in the mesa region, and a modal kinetic energy ratio is introduced to characterize the modal coupling. The influence of structural parameters on the spectral characteristics and mode coupling intensity is analyzed. The results indicate that mesa parameters mainly regulate the resonance frequency and energy trapping of the primary thickness-shear mode. In contrast, due to changes in flexure stiffness of the ends and the reflection angle of elastic waves, the beveled edge parameters affect the coupling intensity between the primary thickness-shear mode and parasitic modes. The optimized parameter combinations lead to low coupling states with nearly pure thickness-shear vibration, while the mesa- and bevel-length related low coupling intervals exhibit better fabrication robustness.

## 1. Introduction

Driven by demands for higher integration, lower power consumption, and coordinated multifunctional operations, the sizes of 5G communication modules, wearable electronics, and Internet of Things terminals continue to shrink [1,2]. This trend places growing requirements on the miniaturization and frequency stability of frequency control components [3,4,5,6]. AT-cut quartz crystal resonators (QCRs) operating in the fundamental thickness-shear mode remain widely used in communication modules, sensing devices, and high-precision electronic systems owing to their high-quality factor, stable frequency–temperature characteristics, and mature manufacturing processes [7,8,9]. However, as the size of the resonator decreases, the influence of edge geometry and its boundary effects on vibration modes becomes increasingly significant. These effects reduce the modal separation between the fundamental thickness-shear mode and adjacent spurious modes and increase the risk of mode coupling. This limits further improvements in frequency stability and energy trapping performance [8,10].

As AT-cut QCRs structures are miniaturized, their performance grows increasingly sensitive to the wafer thickness, edge profile and local fabrication accuracy. Conventional grinding and polishing processes have inherent limitations in batch consistency, size control, and the fabrication of complex local structures. The manufacturing requirements for miniaturized QCRs cannot be fully satisfied [4]. Microfabrication techniques, including photolithography assisted wet etching, deep reactive ion etching and laser frequency trimming, have thus been adopted to form inverted mesa structures, locally thinned regions and non-uniform thickness profiles [11,12,13,14,15]. Nevertheless, the etching behavior of quartz is strongly dependent on crystallographic orientation. The difference in the etch rate across crystal planes leads to inclined sidewalls and edge bevels during the wet etching process [16,17,18]. The quality of the sidewalls can be improved by adjusting the etching system, modifying mask patterns, and optimizing process parameters. Even so, the beveled edges caused by anisotropic etching cannot be fundamentally eliminated [13,19]. For large-sized resonators, such beveled edges only occupy a small fraction of the entire structure, and their impact on the main vibration region can be ignored. In contrast, for miniature devices, the edge region occupies a much larger relative proportion. Accordingly, the local thickness, stiffness, and boundary reflection conditions are altered significantly [10].

Existing research on energy trapping and spurious mode suppression in AT-cut QCRs has addressed structural optimization from multiple perspectives, including crystal plate thickness distribution, electrode configuration and boundary design. For mesa-type multichannel quartz crystal microbalances, Shen et al. [20] found that local thickness variations in the crystal plate enhance the confinement of thickness-shear vibrations within the active region and reduce the frequency interference between adjacent channels. Lu et al. [21] employed the finite element method to analyze double stepped mesa structures, revealing that a centrally thinned region, combined with an outer thick supporting zone, enhances energy trapping performance. He et al. [22] investigated thickness-shear and torsional coupling vibrations in AT-cut QMRs and reported that mesa step boundaries restrict energy leakage while altering the local wave reflection process. Electrode design offers an alternative approach to tuning energy trapping. Partial electrodes, ring electrodes and lateral field excitation all improve modal selectivity by altering mass loading patterns and electric field distributions [23,24,25]. Continuously contoured structures, such as hyperbolic, elliptical, and Gaussian profiles, reduce abrupt boundary changes by providing smooth thickness transitions, thereby reducing stress and electric field concentration and suppressing spurious modes [26,27,28,29]. Collectively, these studies indicate that geometric configuration determines the strength of energy trapping and modulates the coupling between the thickness-shear mode and adjacent modes.

Numerical analysis is an essential tool for interpreting the evolution of frequency spectra and the mechanisms of mode coupling in resonators with complex geometries. Sun et al. [10] developed a partition geometric fitting approach for beveled crystal plates, in which regions of non-uniform thickness are discretized into multiple subdomains to obtain approximate solutions. This method widens the applicability of high-frequency vibration analysis to resonators with sophisticated edge architectures. Li et al. [27] investigated continuously contoured AT-cut quartz plates by establishing an anti-plane vibration model and solving energy trapped thickness-shear modes via power series expansion. Their research results indicate that the geometric non-uniformity has a pronounced effect on resonance frequencies, mode shapes, and shear-wave propagation characteristics. Lee and Wang [30] established a two-dimensional model for coupled thickness-shear and flexural vibrations of contoured crystal strips and obtained power series solutions for linearly varying thickness regions using the Frobenius method. Wang et al. [31] further improved the numerical accuracy of the eigenfrequencies and corresponding mode shapes through multiprecision computation. These studies revealed the regulatory role of thickness profiling in thickness-shear and flexural mode coupling. Li et al. [32] examined energy trapping events in rectangular AT-cut QCRs by adopting the variational form of the first-order Mindlin plate equations and combining it with the Ritz method, revealing that finite plate sizes and local electrode deposition can modify the spatial distribution of thickness-shear modes. Li et al. [33,34] further explored the effects of plate parallelism errors and surface roughness on coupled vibration responses, demonstrating that even small geometric deviations can cause changes in frequency and mode shape. The existing research has systematically revealed the effects of variable thickness structures, electrode perturbations, and fabrication errors on the vibrational characteristics of the AT-cut QCRs. However, for micro QMRs incorporating both mesas and etched bevels, the influence of geometric nonuniformity on modal coupling has not yet been systematically clarified. As device dimensions continue to decrease, bevel-induced edge effects may increasingly alter the interaction between the primary mode and adjacent parasitic modes. Therefore, it is necessary to further elucidate how the mesa geometry and bevel parameters govern the coupling behavior.

This article focuses on the beveled edge structures formed by photolithography etching processes in the micro AT-cut QMRs. Taking the beveled edge region into account, a vibration analysis model of the QMRs is established and validated against finite element results. The effects of the mesa structure and beveled edges on the coupling between the primary thickness-shear mode and flexural spurious modes is investigated by analyzing the frequency spectrum characteristics and mode coupling behavior under different structural parameters. The research results can provide references for the structural design and performance optimization of the micro AT-cut QMRs.

## 2. Theoretical Model and Analytical Solution

### 2.1. AT-Cut QMR Model with Beveled Edges

**Figure 1:**
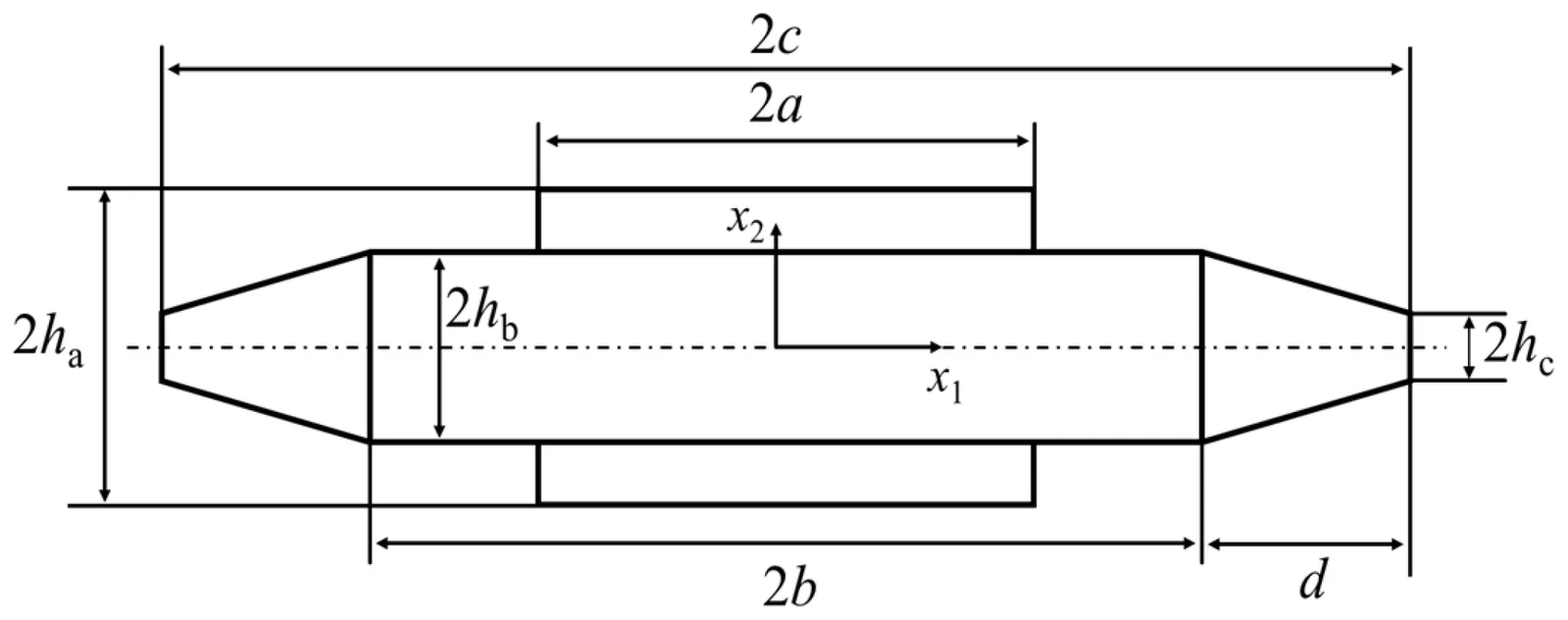
Schematic diagram of an AT-cut QMR with beveled edges.

Figure 1 shows the AT-cut QMR, which is symmetric about the mid-planes *x*_1_ = 0 and *x*_2_ = 0. The resonator consists of a quartz plate with a stepped thickness distribution, and its outer edge is beveled. A straight crested wave approximation is adopted along the *x*_3_-direction. Three-dimensional dispersion analysis shows that the first order thickness-shear (denoted by TSh-1) mode remains well isolated for width-to-thickness ratios of 10.22 to 10.36, supporting the validity of the present two-dimensional approximation within this range [35,36]. Owing to the symmetry about the plane *x*_1_ = 0, only the right half of the resonator is analyzed. According to the thickness distribution and geometric profile, the right half-domain is divided into three regions: the central mesa region *V*_a_, the outer flat base region *V*_b_, and the beveled edge region *V*_c_. The corresponding interfaces and surface are located at *a*, *b*, and *c*, where *a* < *b* < *c*.

For the right half-domain, the local half-thickness of the crystal plate is denoted by *h*(*x*_1_). From the piecewise geometric variation of the thickness along the *x*_1_-direction in the right half of the structure, the following relation is obtained.

(1) $$h\left(x_{1}\right)=\left\{\begin{array}{c} h_{a},\;0\leq x_{1}\leq a\\ h_{b},\;a\leq x_{1}\leq b\\ h_{b}\left(1-\xi X\right),\;b\leq x_{1}\leq c \end{array}\right.$$

where

(2) $$h_{e}=h_{a}-h_{b},\;\xi =1-\frac{h_{c}}{h_{b}},\;X=\frac{x_{1}-b}{d},\;0\leq X\leq 1$$

where *h*_a_ and *h*_b_ denote the half-thicknesses of the mesa area and the flat base region, respectively. *h*_e_ denotes the half-thickness difference between the mesa region and the flat base region, *h*_c_ is the half-thickness at the free edge, *ξ* is the thickness reduction coefficient of the beveled edge region, with 0 ≤ *ξ* < 1, and the length of the beveled edge region *d* = *c* − *b*.

The coupling vibration between the thickness-shear force and flexure of the QMR can be described by the first-order Mindlin plate theory [36,37]. The displacement field is assumed as follows.

(3) $$u_{1}\left(x_{1},x_{2},t\right)=x_{2}u\left(x_{1}\right)e^{i\omega t},\;u_{2}\left(x_{1},x_{2},t\right)=w\left(x_{1}\right)e^{i\omega t}$$

where *u*_1_ and *u*_2_ denote the displacement components along the *x*_1_- and *x*_2_-directions, respectively; *u*(*x*_1_) is the generalized thickness-shear rotation; *w*(*x*_1_) is the transverse displacement of the plate mid-plane. *ω* is the angular frequency, i is the imaginary unit, and *t* is time.

According to the first-order Mindlin plate theory for coupling vibration between thickness-shear and flexure, the governing equations for steady-state free vibration are

(4) $$T_{6,1}^{\left(0\right)}+2\rho h\omega^{2}w=0$$

(5) $$T_{1,1}^{\left(1\right)}-T_{6}^{\left(0\right)}+\frac{2\rho h^{3}}{3}\omega^{2}u=0$$

where *ρ* is the material density, $T_{6}^{\left(0\right)}$ and $T_{1}^{\left(1\right)}$ with $T_{i}^{\left(j\right)}=\int_{-h}^{h}T_{i}x_{2}^{\left(j\right)}dx_{2}$ are the thickness-shear force and the flexure moment per unit width, respectively. A comma followed by 1 indicates taking the derivative of *x*_1_. The corresponding constitutive relations are

(6) $$T_{6}^{\left(0\right)}=2\kappa^{2}hC_{66}\left(w_{,1}+u\right)$$

(7) $$T_{1}^{\left(1\right)}=\frac{2h^{3}}{3}{\tilde{C}}_{11}u_{,1}$$

where ${\bar{C}}_{11}=C_{11}-C_{12}^{2}/C_{22}$. *C*_11_, *C*_12_, *C*_22_, and *C*_66_ are the elastic constants of the AT-cut quartz; The shear correction factor $\kappa^{2}=\pi^{2}/12$.

Combining Equations (4)–(7) yields the coupled differential equations for *u* and *w*

(8) $$\begin{array}{l} -3\kappa^{2}C_{66}\left(w_{,1}+u\right)+3{\bar{C}}_{11}hh_{,1}u_{,1}+{\bar{C}}_{11}h^{2}u_{,11}+\rho \omega^{2}h^{2}u=0\\ \kappa^{2}C_{66}\left[h_{,1}\left(w_{,1}+u\right)+h\left(w_{,11}+u_{,1}\right)\right]+\rho \omega^{2}hw=0 \end{array}$$

From Equation (8), the following fourth-order governing equation for *u* is obtained.

(9) $$R_{,11}-\frac{h_{,1}}{h}R_{,1}+\frac{\rho \omega^{2}}{\kappa^{2}C_{66}}R-2\rho h\omega^{2}u=0$$

where $R=\frac{2}{3}{\bar{C}}_{11}{\left(h^{3}u_{,1}\right)}_{,1}+\frac{2\rho h^{3}}{3}\omega^{2}u$. For the mesa region and the flat base region, the local half thickness *h*(*x*_1_) of the crystal plate is a constant; while for the beveled edge region, *h*(*x*_1_) is a linear function.

### 2.2. Partition Analytical Solution and Characteristic Frequency Equation

For any uniform thickness region *j* (*j* = a, b) of the crystal plate, the half-thickness is denoted by *h_j_*. To introduce a common frequency normalization, the global dimensionless frequency is defined with respect to the thickness-shear reference angular frequency $\Omega =\omega /\omega_{a}^{*}$ of the mesa region.

(10) $$\omega_{a}^{*}=\frac{\pi }{2h_{a}}\sqrt{\frac{C_{66}}{\rho }}$$

where $\omega_{a}^{*}$ is the thickness-shear reference frequency of the mesa region. The local normalized frequency is then calculated as

(11) $$\Omega_{j}=\frac{h_{j}}{h_{a}}\Omega$$

Substituting the half-thickness *h_j_* of the uniform thickness region into Equation (9), the fourth-order differential equation with constant coefficient is obtained.

(12) $$\chi h_{j}^{4}u_{j,1111}+3h_{j}^{2}\Omega_{j}^{2}\left(1+\chi \right)u_{j,11}-9\Omega_{j}^{2}\left(1-\Omega_{j}^{2}\right)u_{j}=0$$

where the stiffness ratio $\chi ={\bar{C}}_{11}/\left(\kappa^{2}C_{66}\right)$. Let $u_{jr}\left(x_{1}\right)=A_{jr}\mathrm{Exp}\left(iZ_{jr}x_{1}/h_{j}\right)$ with an underdetermined constant $A_{jr}$ and characteristic roots $Z_{jr}\left(r=0,2\right)$ [38]. Submitting these to Equation (12), the dispersion equation of the uniform thickness region can be obtained.

(13) $$\chi Z_{jr}^{4}-3\left(1+\chi \right)\Omega_{j}^{2}Z_{jr}^{2}-9\left(1-\Omega_{j}^{2}\right)\Omega_{j}^{2}=0$$

Let $w_{jr}\left(x_{1}\right)=ig_{jr}h_{j}u_{jr}\left(x_{1}\right)$, and the corresponding amplitude ratio $g_{jr}$ can be obtained from Equation (8) for any uniform thickness regions.

(14) $$g_{jr}=\frac{Z_{jr}}{3\Omega_{j}^{2}-Z_{jr}^{2}}$$

#### 2.2.1. Central Mesa Region *V*_a_

Due to the symmetry of the structure about *x*_1_ = 0, the thickness-shear displacement of the mesa area 0 ≤ *x*_1_ ≤ *a* is in the form of an even function, and the transverse displacement is in the form of an odd function. Therefore, the displacement of the mesa area can be expressed by the real parts.

(15) $$\begin{array}{l} u_{a}\left(x_{1}\right)=\sum_{r=0,2}A_{ar}\cos\left(Z_{ar}\frac{x_{1}}{h_{a}}\right)\\ w_{a}\left(x_{1}\right)=\sum_{r=0,2}g_{ar}A_{ar}h_{a}\sin\left(Z_{ar}\frac{x_{1}}{h_{a}}\right) \end{array}$$

Substituting Equation (15) into the constitutive relations gives the corresponding shear stresses in the mesa region as

(16) $$\begin{array}{l} T_{1a}^{\left(1\right)}\left(x_{1}\right)=-\frac{2h_{a}^{2}}{3}{\tilde{C}}_{11}\sum_{r=0,2}A_{ar}Z_{ar}\sin\left(Z_{ar}\frac{x_{1}}{h_{a}}\right)\\ T_{6a}^{\left(0\right)}\left(x_{1}\right)=2\kappa^{2}h_{a}C_{66}\sum_{r=0,2}A_{ar}\left(1+g_{ar}Z_{ar}\right)\cos\left(Z_{ar}\frac{x_{1}}{h_{a}}\right) \end{array}$$

The state vector of the mesa area is defined as

(17) $$y_{a}\left(x_{1}\right)={\left[u_{a},w_{a},T_{6a}^{\left(0\right)},T_{1a}^{\left(1\right)}\right]}^{T}=S_{a}\left(x_{1}\right)A_{a},\;A_{a}={\left[A_{a0},A_{a2}\right]}^{T}$$

where $S_{a}\left(x_{1}\right)$ is a 4 × 2 coefficient matrix, which can be obtained from Equations (15) and (16).

#### 2.2.2. Outer Flat Base Region *V*_b_

For the flat base region *a* ≤ *x*_1_ ≤ *b*, let $\zeta =x_{1}-a$. This region is no longer directly constrained by the symmetry condition at *x*_1_ = 0, and its displacement solution should include both sine and cosine terms.

(18) $$\begin{array}{l} u_{b}\left(\zeta \right)=\sum_{r=0,2}\left[B_{r}^{c}\cos\left(Z_{br}\frac{\zeta }{h_{b}}\right)+B_{r}^{\zeta }\sin\left(Z_{br}\frac{\zeta }{h_{b}}\right)\right]\\ w_{b}\left(\zeta \right)=\sum_{r=0,2}g_{br}h_{b}\left[B_{r}^{c}\sin\left(Z_{br}\frac{\zeta }{h_{b}}\right)-B_{r}^{\zeta }\cos\left(Z_{br}\frac{\zeta }{h_{b}}\right)\right] \end{array}$$

Accordingly, the corresponding internal forces in the flat base area are obtained as

(19) $$\begin{array}{l} T_{1b}^{\left(1\right)}\left(\zeta \right)=\frac{2h_{b}^{2}}{3}{\tilde{C}}_{11}\sum_{r=0,2}Z_{br}\left[-B_{r}^{c}\sin\left(Z_{br}\frac{\zeta }{h_{b}}\right)+B_{r}^{\zeta }\cos\left(Z_{br}\frac{\zeta }{h_{b}}\right)\right]\\ T_{6b}^{\left(0\right)}\left(\zeta \right)=2\kappa^{2}h_{b}C_{66}\sum_{r=0,2}\left(1+g_{br}Z_{br}\right)\left[B_{r}^{c}\cos\left(Z_{br}\frac{\zeta }{h_{b}}\right)+B_{r}^{\zeta }\sin\left(Z_{br}\frac{\zeta }{h_{b}}\right)\right] \end{array}$$

The state vector of the flat base area is defined as

(20) $$y_{b}\left(\zeta \right)={\left[u_{b},w_{b},T_{6b}^{\left(0\right)},T_{1b}^{\left(1\right)}\right]}^{T}=S_{b}\left(\zeta \right)B,\;B={\left[B_{0}^{c},B_{0}^{\zeta },B_{2}^{c},B_{2}^{\zeta }\right]}^{T}$$

where $S_{b}\left(\zeta \right)$ is a 4 × 4 coefficient matrix, which is obtained from Equations (18) and (19).

#### 2.2.3. Beveled Edge Region *V*_c_

From Equation (1), in the beveled edge region *b* ≤ *x*_1_ ≤ *c*, $h\left(x_{1}\right)=h_{b}\left(1-\xi X\right)$. By letting $\tilde{X}=1-\xi X$ and substituting it into Equation (9), a variable-coefficient fourth-order governing equation for $u_{c}\left(X\right)$ is obtained [31].

(21) $${\tilde{X}}^{2}u_{c,\mathrm{XXXX}}-8\xi \tilde{X}u_{c,\mathrm{XXX}}+3\left(4\xi^{2}+\alpha {\tilde{X}}^{2}\right)u_{c,\mathrm{XX}}-\beta \xi \tilde{X}u_{c,X}+\left(\gamma {\tilde{X}}^{2}+\eta \right)u_{c}=0$$

where the subscript *X* denotes differentiation with respect to the dimensionless coordinate *X*. The parameters in Equation (21) are defined as

(22) $$\begin{array}{l} \alpha =\lambda^{2}\left(1+\frac{1}{\chi }\right),\;\beta =3\lambda^{2}\left(\frac{5}{\chi }+3\right)\\ \gamma =\frac{9\lambda^{4}}{\chi },\;\eta =\frac{9\lambda^{2}}{\chi }\left[\xi^{2}-{\left(\frac{d}{h_{b}}\right)}^{2}\right] \end{array}$$

with $\lambda =d\Omega /h_{a}$. By letting $u_{c}\left(X\right)=\sum_{n=0}^{\infty }q_{n}X^{n+s}$, substituting it into Equation (21) gives

(23) $$\begin{array}{l} \sum_{n=0}^{\infty }\left(n+s\right)\left(n+s-1\right)\left(n+s-2\right)\left(n+s-3\right)q_{n}X^{n+s-4}\\ -2\xi \sum_{n=0}^{\infty }\left(n+s-1\right)\left(n+s\right)\left(n+s+1\right)\left(n+s-2\right)q_{n}X^{n+s-3}\\ +\sum_{n=0}^{\infty }\left(n+s\right)\left(n+s-1\right)\left\{\xi^{2}\left[12+\left(n+s-2\right)\left(n+s+5\right)\right]+3\alpha \right\}q_{n}X^{n+s-2}\\ -\sum_{n=0}^{\infty }\xi \left(n+s\right)\left[6\alpha \left(n+s-1\right)+\beta \right]q_{n}X^{n+s-1}\\ +\sum_{n=0}^{\infty }\left\{\xi^{2}\left(n+s\right)\left[3\alpha \left(n+s-1\right)+\beta \right]+\gamma +\eta \right\}q_{n}X^{n+s}\\ -2\gamma \xi \sum_{n=0}^{\infty }q_{n}X^{n+s+1}+\gamma \xi^{2}\sum_{n=0}^{\infty }q_{n}X^{n+s+2}=0 \end{array}$$

The function of Equation (23) is to transform the original variable coefficient differential Equation (21) into an algebraic relation concerning the series coefficients $q_{n}$. Since this equation holds for any variable *X*, the coefficients of each power of *X* must respectively be zero. First, we consider the lowest-power term in Equation (23), which is $X^{s-4}$. This term is contributed solely by the *n* = 0 component in the first term; that is

(24) $$s\left(s-1\right)\left(s-2\right)\left(s-3\right)q_{0}X^{s-4}=0$$

Since $q_{0}\neq 0$, the indicial equation gives *s* = 0, 1, 2, 3, corresponding to four linearly independent power series solutions. Accordingly, the initial coefficients *q_i_* (*i* = 0, 1, 2, 3) are introduced as four independent arbitrary constants. By collecting the coefficients of like powers of *X* in Equation (23) and setting them to zero, the recursive relations for the remaining higher-order coefficients can be obtained.

(25) $$\begin{array}{l} q_{n+6}=2\xi q_{n+5}-\left[\xi^{2}+\frac{3\alpha }{\prod_{k=1}^{2}\left(n+4+k\right)}\right]q_{n+4}+\frac{\xi \left[6\alpha \left(n+2\right)+\beta \right]}{\prod_{k=1}^{3}\left(n+3+k\right)}q_{n+3}\\ -\frac{\xi^{2}\left(n+2\right)\left[3\alpha \left(n+1\right)+\beta \right]+\gamma +\eta }{\prod_{k=1}^{4}\left(n+2+k\right)}q_{n+2}+\frac{2\xi \gamma }{\prod_{k=1}^{4}\left(n+2+k\right)}q_{n+1}-\frac{\xi^{2}\gamma }{\prod_{k=1}^{4}\left(n+2+k\right)}q_{n} \end{array}$$

where n=−2,−1,0,1,…, $q_{-2}=q_{-1}=0$.

Express the four initial arbitrary constants using the four independent amplitude coefficients *C_i_* (*i* = 0, 1, 2, 3) as

(26) $$\begin{array}{l} q_{0}=C_{0}+C_{2},\;q_{1}=C_{1}+C_{3}\\ q_{2}=C_{0}-C_{2},\;q_{3}=C_{1}-C_{3} \end{array}$$

Since Equation (25) is a linear recurrence relation, for *n* > 3, any higher-order coefficient can be expressed as

(27) $$q_{n}=C_{0}f_{0}\left(n\right)+C_{1}f_{1}\left(n\right)+C_{2}f_{2}\left(n\right)+C_{3}f_{3}\left(n\right)$$

where $f_{i}\left(n\right)$ (*i* = 0, 1, 2, 3) denotes the *n*-th series coefficient function associated with the independent amplitude coefficient $C_{i}$. For *n* = 0, 1, 2, 3, $f_{i}\left(n\right)$ is determined by the coefficient multiplying $C_{i}$ in Equation (27). For *n* > 3, $f_{i}\left(n\right)$ is obtained successively from the recurrence relation in Equation (25). Here,

(28) $$f_{i}\left(-2\right)=f_{i}\left(-1\right)=0,\;i=0,1,2,3$$

By substituting Equations (26) and (27) into the power series solution, the displacement solution in the beveled edge region can be written as

(29) $$u_{c}\left(X\right)=\sum_{i=0}^{3}C_{i}W_{i}\left(X\right)$$

where four linearly independent basis functions *W_i_*(*X*) can be written as

(30) $$W_{i}\left(X\right)=\sum_{n=0}^{\infty }f_{i}\left(n\right)X^{n},\;i=0,1,2,3$$

Substituting Equation (29) into Equations (6)–(8), the internal forces and transverse displacement in the beveled edge region are obtained as

(31) $$\begin{array}{l} T_{1c}^{\left(1\right)}\left(X\right)=\sum_{i=0}^{3}C_{i}M_{i}\left(X\right)\\ T_{6c}^{\left(0\right)}\left(X\right)=\sum_{i=0}^{3}C_{i}Q_{i}\left(X\right)\\ w_{c}\left(X\right)=\sum_{i=0}^{3}C_{i}L_{i}\left(X\right) \end{array}$$

where

(32) $$\begin{array}{l} M_{i}\left(X\right)=\frac{2h_{b}^{3}}{3d}{\bar{C}}_{11}{\tilde{X}}^{3}W_{i,X}\left(X\right)\\ Q_{i}\left(X\right)=\frac{1}{d}M_{i,X}\left(X\right)+\frac{2\rho h_{b}^{3}}{3}{\tilde{X}}^{3}\omega^{2}W_{i}\left(X\right)\\ L_{i}\left(X\right)=-\frac{1}{2d\rho h_{b}\tilde{X}\omega^{2}}Q_{i,X}\left(X\right) \end{array}$$

Thus, the state vector in the beveled edge region can be expressed as

(33) $$y_{c}\left(X\right)={\left[u_{c},w_{c},T_{6c}^{\left(0\right)},T_{1c}^{\left(1\right)}\right]}^{T}=S_{c}\left(X\right)C,\;C={\left[C_{0},C_{1},C_{2},C_{3}\right]}^{T}$$

where $S_{c}\left(X\right)$ is a 4 × 4 coefficient matrix, which can be obtained from Equations (29) and (31).

For the mesa crystal plate shown in Figure 1, which is symmetric with respect to the mid-plane *x*_1_ = 0, continuity conditions must be satisfied at different interfaces. At the interface, *x*_1_ = *a*, between the mesa region and the flat base region, the continuity condition becomes

(34) $$S_{a}\left(a\right)A-S_{b}\left(0\right)B=0$$

At the interface *x*_1_ = *b* between the flat base region and the beveled edge region, one has

(35) $$S_{b}\left(b-a\right)B-S_{c}\left(0\right)C=0$$

As a free boundary at the right end *x*_1_ = *c*, the internal forces satisfy

(36) $$T_{6c}^{\left(0\right)}\left(1\right)=0,\;T_{1c}^{\left(1\right)}\left(1\right)=0$$

Combining Equations (34)–(36) yields the following homogeneous system of linear equations for the unknown coefficients A, B, and C.

(37) $$\left[\begin{array}{ccc} S_{a}\left(a\right) & -S_{b}\left(0\right) & 0\\ 0 & S_{b}\left(b-a\right) & -S_{c}\left(0\right)\\ 0 & 0 & PS_{c}\left(1\right) \end{array}\right]\left[\begin{array}{c} A\\ B\\ C \end{array}\right]=0$$

where $P=\left[\begin{array}{cccc} 0 & 0 & 1 & 0\\ 0 & 0 & 0 & 1 \end{array}\right]$.

By setting the determinant of the coefficient matrix in Equation (37) to zero, the characteristic frequency equation of the mesa crystal plate with beveled edges can be obtained.

## 3. Numerical Results and Discussion

### 3.1. Finite Element Model and Validation

As a benchmark example, an AT-cut QMR with structural parameters *h*_e_/*h*_b_ = 0.1, *a*/*h*_b_ = 9, *ξ* = 0.95, *c*/*h*_b_ = 17, *d*/*h*_b_ = 1 is selected for analysis. Unless otherwise specified in the following sections, these parameters are fixed in the calculations.

**Figure 2:**
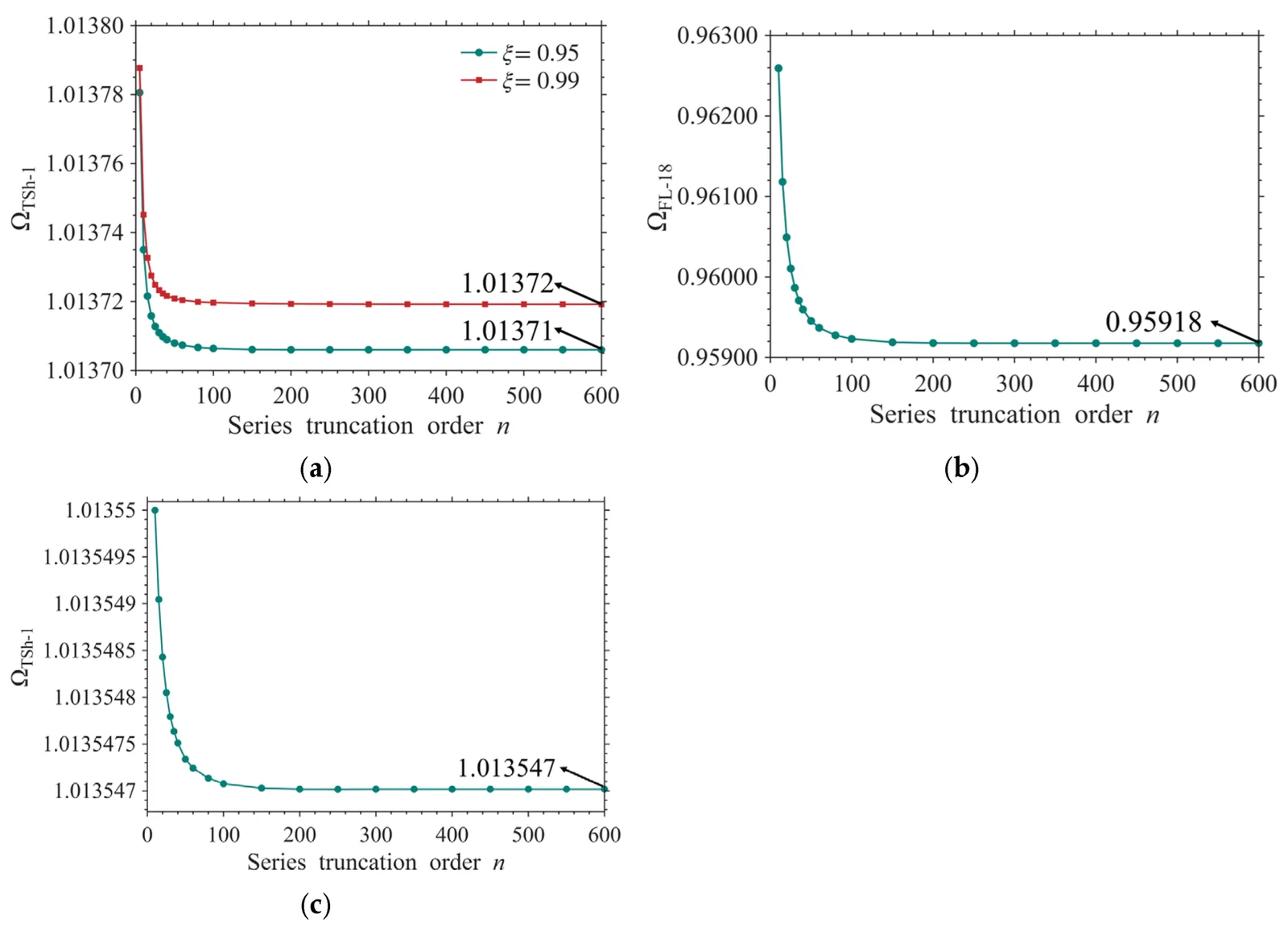
Truncation convergence of the power series solution under different representative conditions: (**a**) TSh-1 mode at *c*/*h*_b_ = 17 for *ξ* = 0.95 and 0.99; (**b**) FL-18 mode at *c*/*h*_b_ = 17; and (**c**) TSh-1 mode in the vicinity of modal anti-crossing at *c*/*h*_b_ = 18.45.

Figure 2 shows the truncation convergence behavior of the power series solution under different representative conditions. Figure 2a compares the frequency convergence of the TSh-1 mode at *c*/*h*_b_ = 17 for *ξ* = 0.95 and the extreme bevel condition *ξ* = 0.99. With increasing *ξ*, the frequency variation at low truncation orders becomes more pronounced. Compared to that at *ξ* = 0.95, convergence at *ξ* = 0.99 requires a smaller truncation order. Nevertheless, as the truncation order *n* increases, both frequencies gradually stabilize and converge to 1.01372 and 1.01371, respectively. Figure 2b presents the convergence behavior of the FL-18 flexural branch. Although relatively large frequency variations are observed at low truncation orders, the calculated frequency gradually converges to approximately 0.95918. Figure 2c further examines the TSh-1 mode in the anti-crossing region at *c*/*h*_b_ = 18.45, where the frequency eventually stabilizes at approximately 1.013547.

For all three representative cases, the frequency variation becomes very small when *n* is between 150 and 200. These results demonstrate that the adopted power-series solution exhibits stable convergence under extreme bevel condition, for flexural modes, and in modal anti-crossing region. Therefore, a truncation order of *n* = 200 is adopted in all subsequent calculations.

To validate the theoretical model, a two-dimensional finite element model of the right half of the resonator is established in software Comsol 6.4, which is consistent with the geometry shown in Figure 1. The reference half-thickness is set to *h*_b_ = 35 μm, and the remaining geometric dimensions are scaled accordingly. The material constants of 35.25° AT-cut quartz are cited from Refs. [35,36], which are consistent with those used in the theoretical analysis. At the symmetry plane *x*_1_ = 0, the anti-symmetry boundary condition is imposed, i.e., tangential displacement *u*_2_ = 0. All other external boundaries are traction-free. The computational domain is discretized using free triangular elements, and the relative tolerance of the solver is set to 10^−6^.

Table 1 lists the mesh convergence results for the TSh-1 mode at *c*/*h*_b_ = 14.75 and 18.45. As the mesh is refined, the corresponding dimensionless frequencies converge to 1.01407 and 1.01374, respectively. The relative differences between the extra fine and extremely fine meshes are only 0.0002% and 0.00197%, which confirm satisfactory mesh convergence. Considering both numerical accuracy and computational efficiency, the extra fine mesh is adopted in the subsequent analyses.

**Figure 3:**
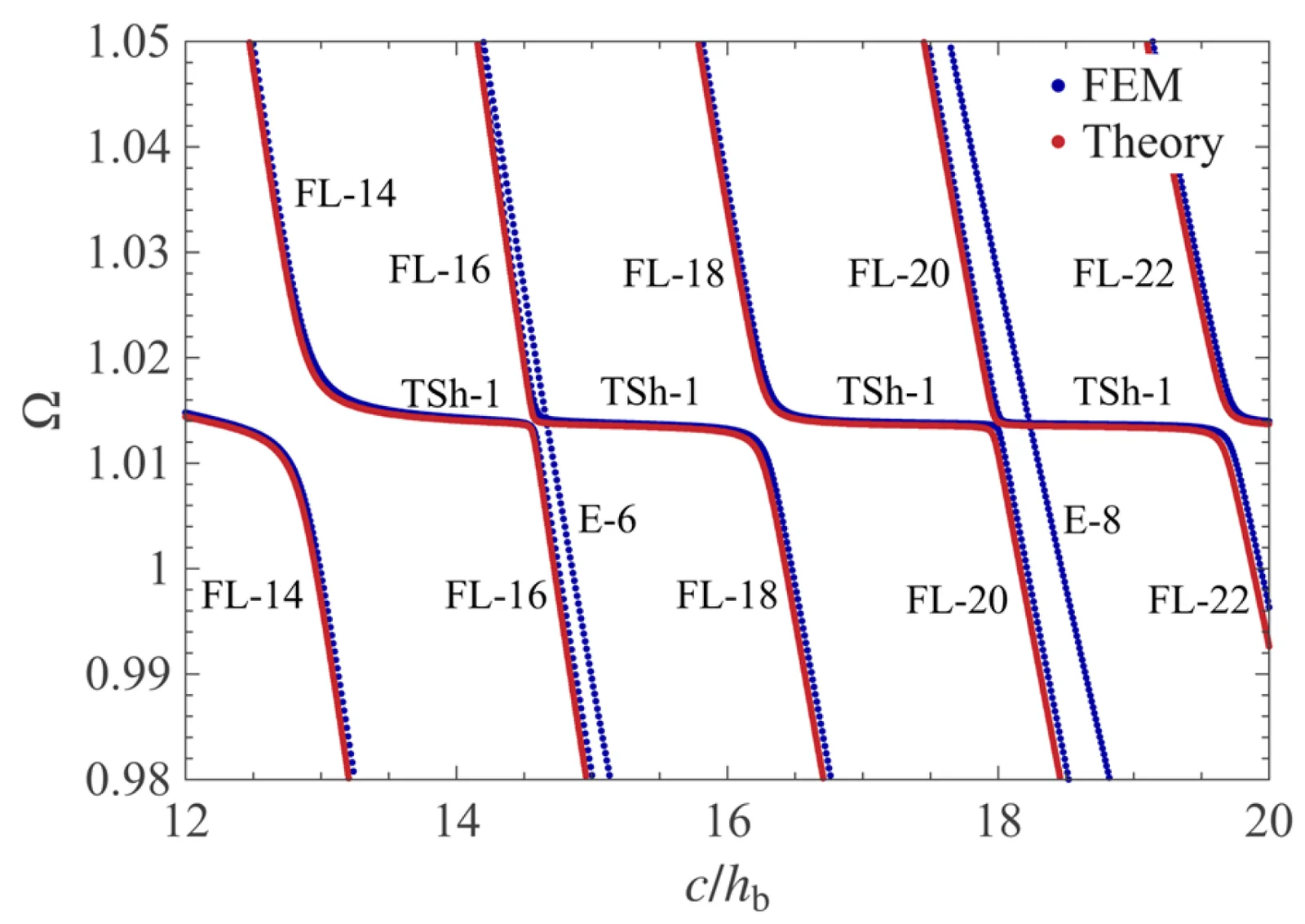
Comparison of the frequency spectra obtained from the theoretical analysis and FEM.

To verify the reliability of the aforementioned solution method, Figure 3 compares the frequency spectra obtained from the theoretical analysis and the finite element method (FEM). The frequencies and evolution trends of the flexural mode branches FL-14, FL-16, FL-18, FL-20, and FL-22, as well as the fundamental thickness-shear mode branch TSh-1, show good agreement between the two methods. The number after the abbreviation of the modal name indicates the modal order and the half-wave number.

Table 2 lists the frequency errors for each branch between theoretical and FEM results, where 12 ≤ *c*/*h*_b_ ≤ 20. The average and maximum relative errors of the TSh-1 branch are 0.04% and 0.20%, respectively. For the FL-14, FL-16, FL-18, FL-20, and FL-22 branches, the average relative errors range from 0.20% to 0.28%. Moreover, the maximum relative errors of all the major frequency branches remain below 0.40%. The discrepancies between the two results are mainly observed in the modal anti-crossing regions and can be attributed to the order reduction approximations introduced in the first-order Mindlin plate theory. Although the FEM can capture the extension mode branches of E-6 and E-8 in modal analysis, it is worth noting that the extension vibration mode in the *x*_1_ direction of the QMR will not be excited when subjected to electric field excitation in the thickness direction. Therefore, the theoretical model does not consider the extension vibration mode.

Table 3 lists the representative FEM calculated eigenfrequencies and corresponding mode shapes for two selected structure ratios. The TSh-1 mode is dominated by thickness-shear vibration localized in the central mesa region, whereas the FL modes exhibit pronounced flexural characteristics. In contrast, E-6 and E-8 correspond to extensional modes that are not included in the present theoretical formulation. Within 12 ≤ *c*/*h*_b_ ≤ 20 and the fundamental thickness-shear resonance frequency range of approximately 0.98 ≤ Ω ≤ 1.03, the TSh-1 and FL-14–FL-22 branches show good agreement between the theoretical and FEM results, supporting the quantitative applicability of the present model within this range and providing a reliable basis for the subsequent analysis of spectral evolution and modal coupling.

### 3.2. Frequency Spectrum and Thickness-Shear Displacement Distribution

**Figure 4:**
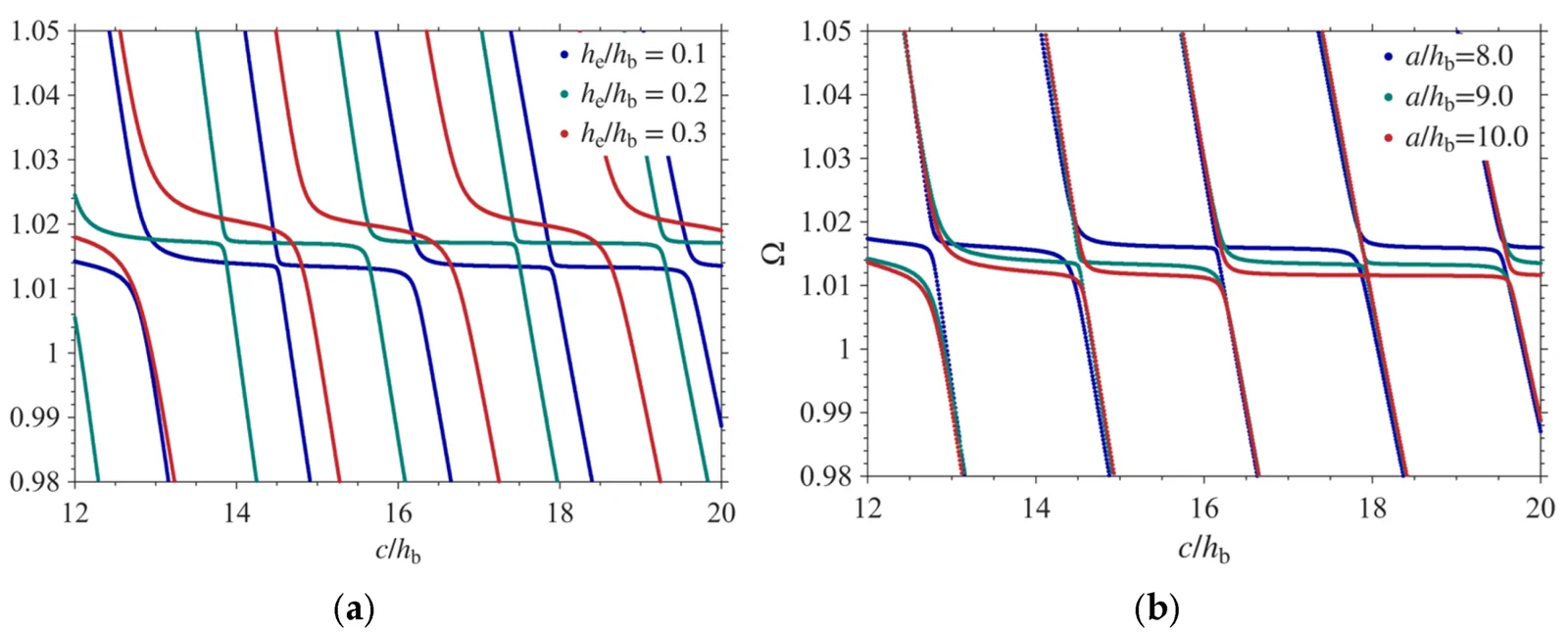
Characteristic frequencies versus length-to-thickness ratios of the AT-cut QMR for different mesa parameters: (**a**) height ratios *h*_e_/*h*_b_; (**b**) length ratios *a*/*h*_b_.

**Figure 5:**
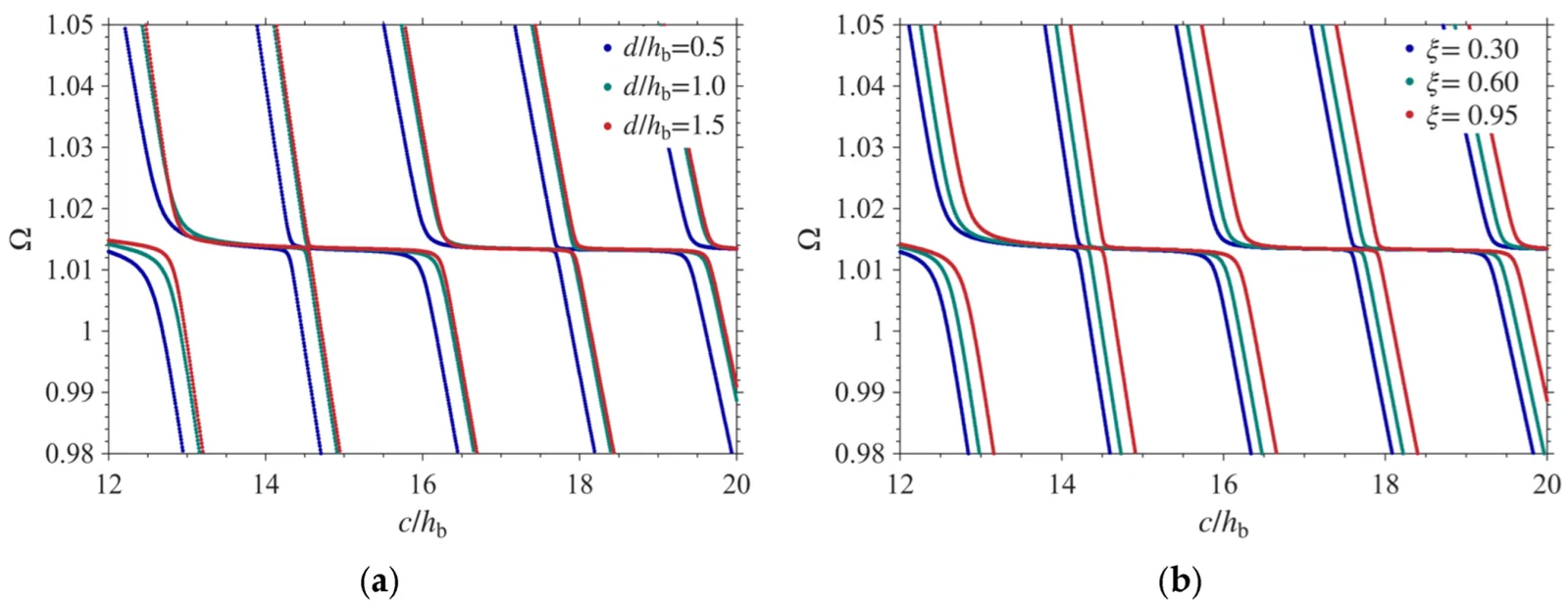
Characteristic frequencies versus length-to-thickness ratios of the AT-cut QMR for different bevel region parameters: (**a**) length ratios *d*/*h*_b_; (**b**) thickness reduction coefficients *ξ*.

To investigate the influence of structural parameters on the thickness-shear mode of the AT-cut QMR, Figure 4 and Figure 5 present the frequency spectra of the dimensionless frequency Ω versus the total length-to-thickness ratio *c*/*h*_b_ for different structural parameters. In these spectra, the nearly horizontal branches correspond to the fundamental thickness-shear mode TSh-1, whereas the inclined branches represent the adjacent flexural mode FL. In Figure 4a, the mesa height ratio *h*_e_/*h*_b_ has the most pronounced effect on the TSh-1 mode. When *h*_e_/*h*_b_ increases from 0.1 to 0.3, the half-thickness of the mesa region increases and the local thickness-shear reference frequency $\omega_{a}^{*}$ decreases, causing the TSh-1 frequency branch to shift toward higher values of Ω. Meanwhile, the FL branch also changes its position, and several crossing points move toward smaller values of *c*/*h*_b_. Figure 4b shows that the mesa half-length ratio *a*/*h*_b_ affects the frequency of the TSh-1 mode. As *a*/*h*_b_ increases from 8.0 to 10.0, the effective vibration range of the primary mode of thickness-shear in the central mesa area expands, and the local area of modal energy increases. At this time, the equivalent inertia mass participating in the vibration increases, and the primary mode frequency decreases, resulting in an overall shift of the TSh-1 branch toward lower frequencies. In contrast, the FL mode is a global flexure vibration distributed along the *x*_1_ direction, and its wavelength and phase conditions are mainly determined by the total length *c*/*h*_b_ and the end boundary. Consequently, compared with the FL mode, the TSh-1 mode is less sensitive to *a*/*h*_b_.

Figure 5a demonstrates that the TSh-1 branch shifts rightward as the bevel length ratio *d*/*h*_b_ increases. By comparing the FL branch interval between 0.5 and 1.0 with that between 1.0 and 1.5, we can see that that the discrepancies across individual curves gradually narrow, and the branch positions tend to converge toward overlap. This behavior arises from the distinct governing roles of two different structural domains: one is that *d*/*h*_b_ primarily modulates the phase accumulation of flexure waves within the bevel region and the reflection characteristics at the structural edge. The other is that the TSh-1 mode is strongly localized in the central mesa region, with its propagation dominated by the local mesa thickness. Accordingly, the variations in the bevel length have almost no effect on the frequency of the TSh-1 mode.

Figure 5b illustrates that the frequency branches shift to the right as the thickness reduction coefficient *ξ* of the beveled region increases from 0.30 to 0.95. The TSh-1 frequency changes only slightly, indicating that the edge thickness has a limited direct influence on the TSh-1 branch. However, the branch position is shifted to the right, indicating that the beveled edge will alter the geometric size required to meet the energy-trapping condition. In contrast, the FL branch exhibits a larger shift and is more sensitive to *ξ*. As *ξ* increases, the thickness of the free end decreases, the local stiffness of the beveled edge region decreases, and the propagation, reflection and phase accumulation conditions of the wave in the edge region all change. In addition, as *ξ* changes, the position of the FL branch is readjusted, and the position and degree of proximity between the two modes also change, which further affects the coupling behavior between the primary mode and the parasitic mode.

**Figure 6:**
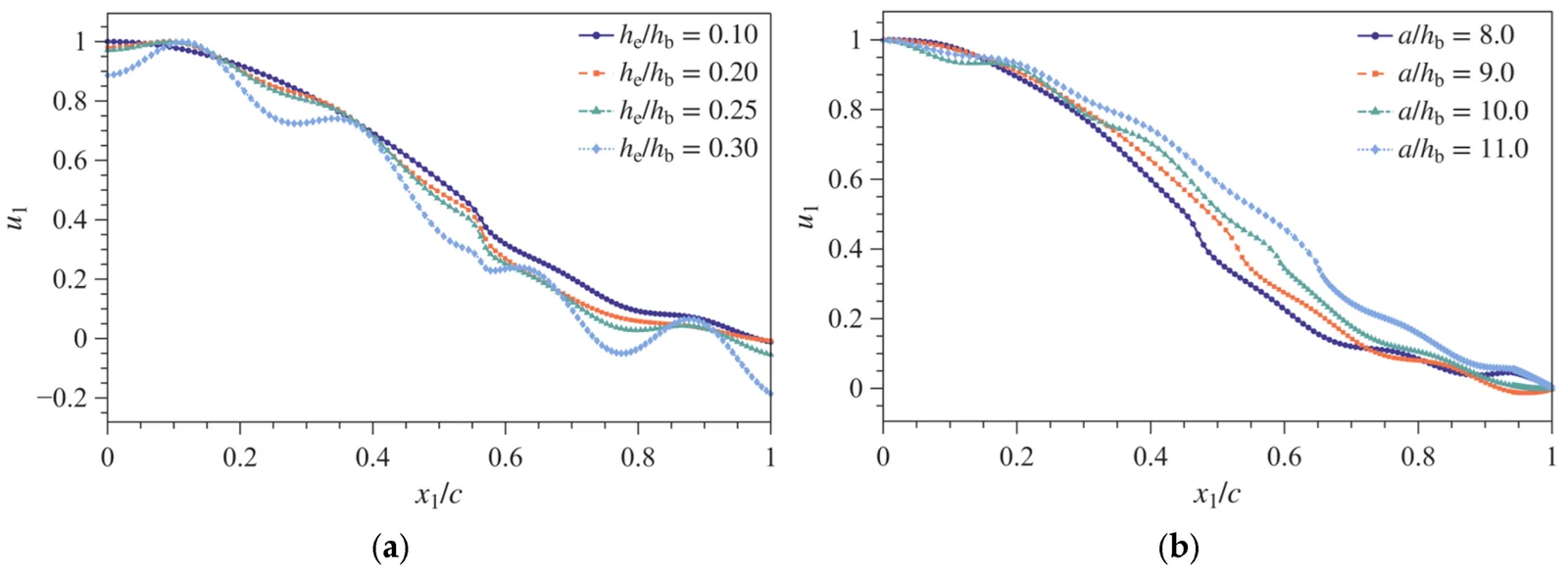
Thickness-shear displacement distribution of the TSh-1 mode for different mesa structural parameters: (**a**) height ratios *h*_e_/*h*_b_; (**b**) length ratios *a*/*h*_b_.

Figure 6 shows the distribution of the thickness-shear displacement *u*_1_ of the TSh-1 mode along *x*_1_/*c* under different mesa structural parameters. All curves reach their maximum values at the middle of the mesa, i.e., *x*_1_/*c* = 0, then decay gradually along the length direction and approach zero near the free edges.

In Figure 6a, as the mesa height ratio *h*_e_/*h*_b_ increases, the displacement fluctuations in the flat base and edge regions become progressively more pronounced. In particular, when *h*_e_/*h*_b_ = 0.3, as shown in Figure 4a, the TSh-1 mode enters the interaction region between the thickness-shear and flexural modes with enhanced modal coupling. Consequently, the local oscillations in the displacement curve become more pronounced, and its smoothness is reduced. As indicated by Equations (6) and (7), the half-thickness of the mesa region directly enters the shear and flexure terms of the governing equations. A larger mesa height amplifies the stiffness mismatch between the mesa and the flat base, leading to stronger wave reflection at the interface. This enhanced reflection weakens the TSh-1 mode confinement in the central mesa region, while making the outer regions more susceptible to local oscillations arising from thickness-shear and flexure coupling.

In Figure 6b, when the mesa length ratio *a*/*h*_b_ increases from 8.0 to 11.0, the high amplitude displacement region extends outward, and the decay position shifts toward the edge. This behavior is associated with the variation in the phase term *Z*_a*r*_*a*/*h*_a_ in Equation (15) caused by increasing *a*/*h*_b_, which modifies the interface continuity conditions in Equation (34). As a result, the effective propagation range of the thickness-shear wave in the central mesa region is enlarged. For the TSh-1 mode, a high amplitude is maintained over a larger area, displacement attenuation slows, and energy trapping is enhanced.

**Figure 7:**
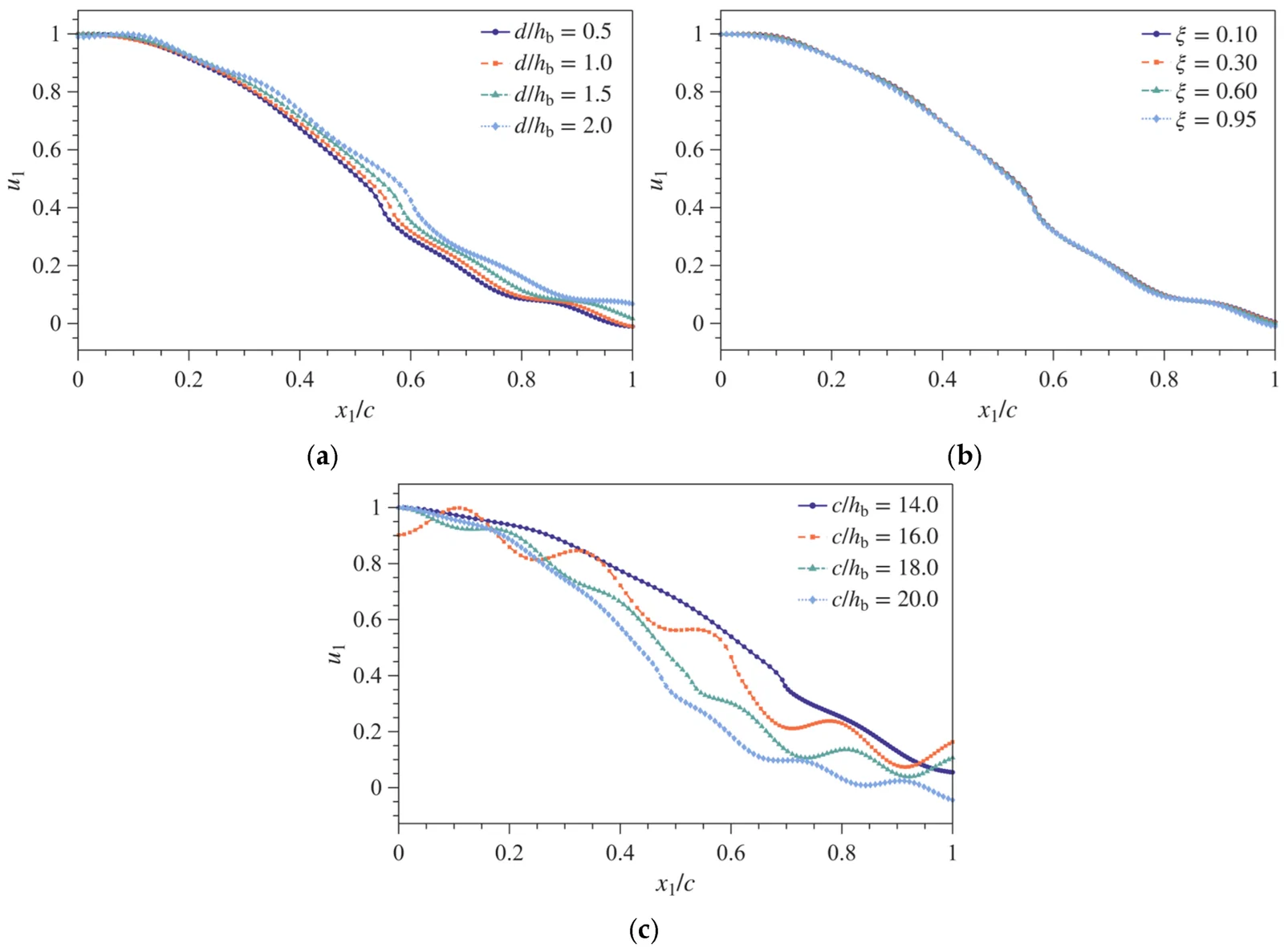
Thickness-shear displacement distribution of the TSh-1 mode for different parameters of the beveled edge region and total length: (**a**) length ratios *d*/*h*_b_; (**b**) thickness reduction coefficients *ξ*; and (**c**) total length ratios *c*/*h*_b_.

Figure 7 illustrates the influence of the beveled edge parameters and the overall length on the thickness-shear displacement *u*_1_ distribution of the TSh-1 mode.

In Figure 7a, as the length ratio *d*/*h*_b_ of the beveled edge region changes, the displacement curve changes noticeably in the edge region, whereas the displacement in the central mesa region remains nearly unchanged. The TSh-1 mode energy is mainly confined within the central mesa region, and its dominant vibration characteristics are governed by the mesa thickness and the continuity conditions at the interfaces. Therefore, the change in the length of the edge bevel shear zone only indirectly affects the TSh-1 mode through weak feedback so that it is difficult to significantly alter the displacement distribution in the central region.

In Figure 7b, the displacement curves nearly overlap as the bevel thickness reduction coefficient *ξ* increases from 0.10 to 0.95. Because *ξ* primarily alters the thickness gradient near the edge, while the TSh-1 displacement has already decayed substantially before reaching the beveled region, variations in *ξ* have only a weak influence on the central mesa region. Consequently, the overall displacement distribution remains almost unchanged.

In Figure 7c, as the total length *c*/*h*_b_ increases, the fluctuations and downward shifts occur in the middle and rear portions of the displacement curve, whereas the main peak in the central region remains nearly unchanged. A larger *c*/*h*_b_ lengthens the flat base region, which alters the phase accumulation conditions of the outer region and the reflection position of the free end. Consequently, the displacement curve exhibits fluctuations of different amplitudes, especially near the edges.

The results of Figure 7 are consistent with those of Figure 5. For different *d* and *ξ* but fixing *c*/*h*_b_ = 17, the frequencies are all located on the frequency branch with a same value as shown in Figure 5; so, the displacement curve changes little, as shown in Figure 7a,b. However, by fixing *ξ* = 0.95, the frequency point falls at the branch intersections between thickness-shear and flexure when *c*/*h*_b_ = 16, 18, and 20; so, their displacement curves lose the TSh-1 mode trapping effect at *c*/*h*_b_ = 14, as shown in Figure 7c.

### 3.3. Thickness-Shear and Flexural Mode Coupling Characteristics

**Figure 8:**
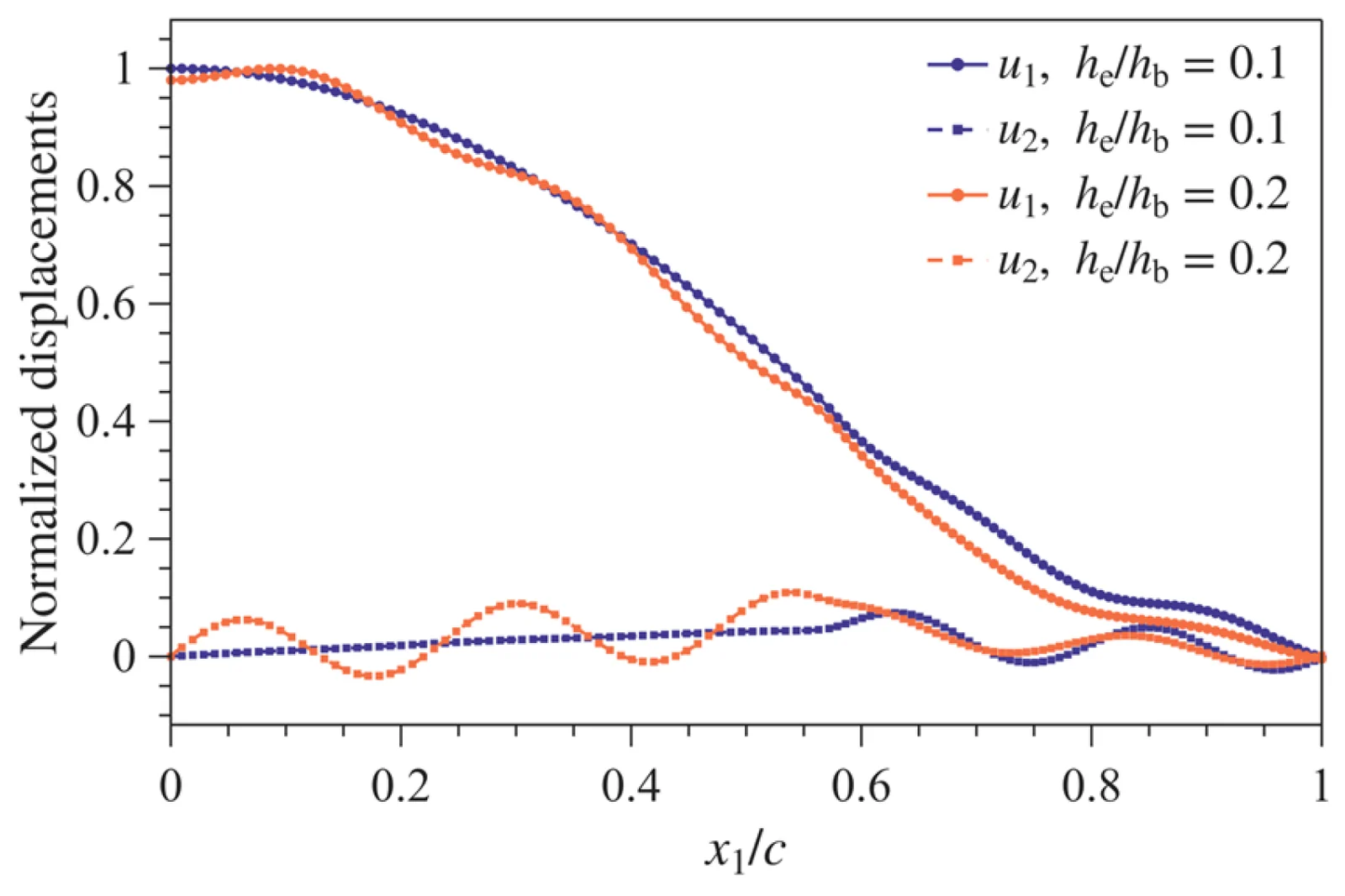
Thickness-shear and flexure displacement distributions of the TSh-1 mode for two mesa height ratios *h*_e_/*h*_b_.

Figure 8 shows the distribution of the TSh-1 modal thickness-shear displacement *u*_1_ and flexural displacement *u*_2_ along *x*_1_/*c* for two mesa height ratios *h*_e_/*h*_b_. The displacement *u*_1_ is the dominant displacement component, reaching its maximum in the central region and gradually decaying towards both ends. Though the amplitude of *u*_2_ is relatively small, exhibiting only weak oscillations, it indicates the presence of a flexural vibration component in the TSh-1 mode. When *h*_e_/*h*_b_ increases from 0.1 to 0.2, the overall distribution of *u*_1_ changes little, while the oscillation amplitude of *u*_2_ increases.

To quantitatively evaluate the relative contributions of the thickness-shear and flexural components, a modal kinetic energy ratio *e*_TSh-1_ in the *x*_1_-direction is defined for the thickness-shear mode [39,40,41]. From Equation (3), the *e*_TSh-1_ of the TSh-1 mode can be calculated as

(38) $$e_{\mathrm{TSh}\text{-}1}=\frac{\int_{V}u_{1}^{2}dV}{\int_{V}\left(u_{1}^{2}+u_{2}^{2}\right)dV}=\frac{\int_{0}^{c}h^{3}\left(x_{1}\right){\left|u\left(x_{1}\right)\right|}^{2}dx_{1}}{\int_{0}^{c}h^{3}\left(x_{1}\right){\left|u\left(x_{1}\right)\right|}^{2}dx_{1}+\;3\int_{0}^{c}h\left(x_{1}\right){\left|w\left(x_{1}\right)\right|}^{2}dx_{1}}$$

It can be seen that the total kinetic energy includes the TSh-1 and FL components. The thickness-shear component dominates the mode when *e*_TSH-1_ approaches unity. Accordingly, *e*_TSh-1_ quantitatively reflects the kinetic energy ratio of TSh-1.

To further clarify modal coupling, a modal coupling index is constructed based on the discrete Fourier spectrum of surface displacement in the mesa region. On the surface of the central mesa, the thickness-shear and flexure displacements are expressed as

(39) $${\tilde{u}}_{1}\left(x_{1}\right)=h_{a}u_{a}\left(x_{1}\right),\;{\tilde{u}}_{2}\left(x_{1}\right)=w_{a}\left(x_{1}\right)$$

Using the symmetry and anti-symmetry conditions of $u_{a}\left(-x_{1}\right)=u_{a}\left(x_{1}\right)$ and $w_{a}\left(-x_{1}\right)=-w_{a}\left(x_{1}\right)$, the displacement distributions are reconstructed over [−a,a] and uniformly sampled at *N* points, yielding discrete sequences ${\tilde{u}}_{l}\left(x_{m}\right)\left(l=1,2;\;m=1,2,\ldots ,N\right)$. To suppress spectral leakage and eliminate the zero-wavenumber component, each sequence is first demeaned by subtracting its sample mean and then weighted by a squared Hanning window $p_{m}$. The preprocessed sequences are given by

(40) $$v_{\mathrm{lm}}=p_{m}\left[{\tilde{u}}_{l}\left(x_{m}\right)-\frac{1}{N}\sum_{p=1}^{N}{\tilde{u}}_{l}\left(x_{p}\right)\right],\;p_{m}=\frac{1}{4}{\left[1-\cos\frac{2\pi \left(m-1\right)}{N-1}\right]}^{2}$$

On this basis, a discrete Fourier transform is performed on the preprocessed sequence, yielding the component-resolved spectral amplitude.

(41) $$H_{l}\left(k\right)=\left|\sum_{m=1}^{N}v_{\mathrm{lm}}e^{-i2\pi k\eta_{m}}\right|$$

where the normalized coordinate in the mesa region $\eta_{m}=\left(x_{m}+a\right)/2a$ with $0\leq \eta_{m}\leq 1$, and *k* is the dimensionless wavenumber. The wavenumbers corresponding to TSh-1 and FL are denoted as *k*_TS-1_ and *k*_FL_, respectively. The mode coupling strength is characterized by the degree of FL-component admixture in the dominant TSh-1 vibration. Thus, the mode coupling strength index is defined as [42]

(42) $$\Gamma =\frac{H_{2}\left(k_{\mathrm{FL}}\right)}{H_{1}\left(k_{\mathrm{TSh}\text{-}1}\right)}$$

The smaller *Γ* is, the weaker the spectral admixture of the FL component in the primary TSh-1 mode. Conversely, the larger *Γ* indicates the more pronounced FL spectral component. Combined with *e*_TSh-1_, the interaction between the two modes can be characterized from the perspectives of modal composition and wavenumber spectrum.

**Figure 9:**
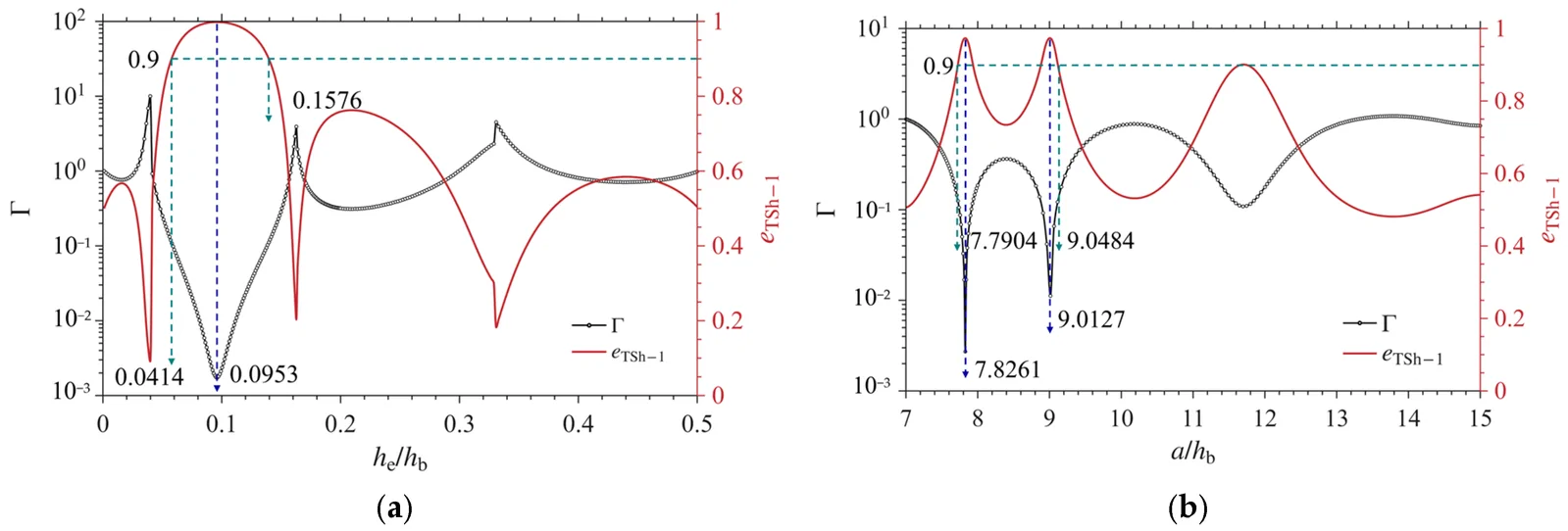
Dependence of the mode coupling index *Γ* and the modal kinetic energy ratio *e*_TSh-1_ on mesa structural parameters: (**a**) height ratios *h*_e_/*h*_b_; (**b**) length ratios *a*/*h*_b_. The data near the dashed line are the coordinate values.

**Figure 10:**
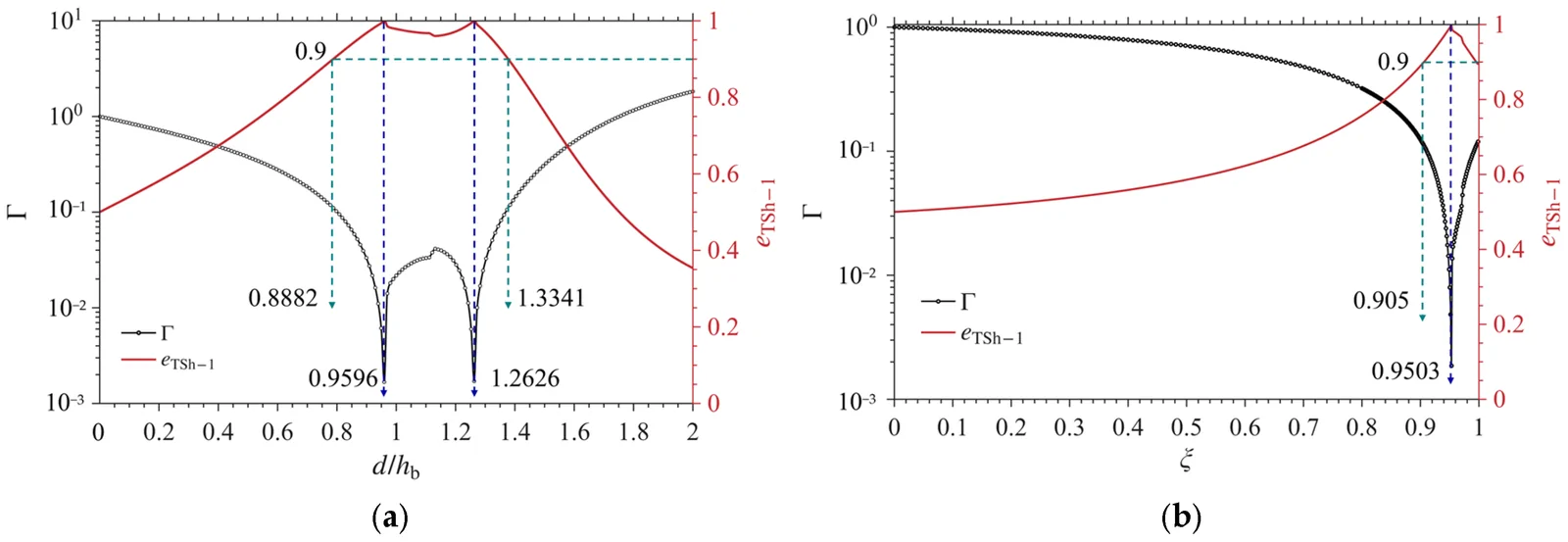
Dependence of the mode coupling index *Γ* and the modal kinetic energy ratio *e*_TSh-1_ on mesa structural parameters: (**a**) length ratios *d*/*h*_b_; (**b**) thickness reduction coefficient *ξ*. The data near the dashed line are the coordinate values.

Figure 9 and Figure 10 show the variations of *Γ* and *e*_TSh-1_ with the mesa structural parameters and beveled-edge parameters, respectively. In Figure 9a, the mesa height ratio *h*_e_/*h*_b_ has a pronounced and non-monotonic influence on the mode coupling strength, with several peaks and valleys appearing in the curve. A minimum value occurs near *h*_e_/*h*_b_ = 0.0953. The mesa height modifies the thickness contrast between the central mesa region and the flat base region, thereby regulating the coupling between the TSh-1 mode and the FL component. The variation in *e*_TSh-1_ is consistent with that of *Γ*. In some parameter intervals, *Γ* decreases, and *e*_TSh-1_ increases, which indicates an enhanced contribution of the TSh-1 component to the FL mode. Near the valleys of *Γ*, the TSh component becomes dominant, whereas the FL component is significantly suppressed. A comparison of the results for *h*_e_/*h*_b_ between 0.1 and 0.2 in Figure 8 and Figure 9a shows that, when *h*_e_/*h*_b_ approaches a valley of the curve of the coupling strength, the oscillation amplitude of the flexure displacement *u*_2_ decreases significantly. The proportion of the flexure component in the thickness-shear primary mode drops accordingly, and the coupling effect weakens in tandem. This variation pattern agrees well with that of the coupling index *Γ*, confirming the validity of the above analysis.

In Figure 9b, the coupling strength *Γ* exhibits an oscillatory variation with increasing *a*/*h*_b_, with pronounced minima appearing at 7.8261 and 9.0127. At these two minima, *e*_TSh-1_ approaches unity, confirming that the corresponding eigenmode is close to a pure TSh vibration. The mesa half-length ratio *a*/*h*_b_ directly modifies the phase term between the mesa region and the flat base region. When this phase condition favors the localization of the dominant TSh-1 component while suppressing the FL component, *Γ* is reduced.

Figure 10a shows two valleys in the coupling-strength curve *Γ* at *d*/*h*_b_ = 0.9596 and 1.2626. At these positions, *e*_TSh-1_ approaches unity, indicating that the FL contribution to the coupling mode is strongly suppressed. The corresponding decrease in the FL spectral component is also reflected by the small *Γ*.

Figure 10b illustrates a pronounced valley of *Γ* near *ξ* = 0.9503. As *ξ* increases, *e*_TSh-1_ generally increases, indicating a reduction in the flexural contribution to the coupling mode. Meanwhile, the edge region becomes sharper, reducing the reflection of flexural waves from the free end. Consequently, less reflected vibration is transmitted back to the central mesa region, leading to a decrease in the FL spectral component of *u*_2_ and thus a reduction in *Γ*.

Some of the coupling minima in Figure 9 and Figure 10 are relatively sharp; since the exponential axis with a base of 10 is applied, a manufacturing tolerance analysis is then performed. Consider the processing capability, a dimensional tolerance of ±2.5 μm is adopted and converted into the corresponding perturbation ranges of the dimensionless structural parameters. Take *e*_TSh-1_ ≥ 0.90 as the criterion for maintaining a TSh-1-dominated low coupling state, the minimum *e*_TSh-1_ within the prescribed tolerance window and the corresponding admissible low coupling interval is determined for each nominal low coupling point, as summarized in Table 4.

For *a*/*h*_b_ = 7.8261 and 9.0127, the minimum *e*_TSh-1_ within the tolerance window are 0.991 and 0.993, respectively. For *d*/*h*_b_ = 0.9596 and 1.2626, the corresponding minima are 0.962 and 0.942. These results indicate that the mesa length and bevel length exhibit good robustness against manufacturing deviations. In contrast, *h*_e_/*h*_b_ = 0.0953 and *ξ* = 0.9503 are more sensitive to machining errors, with the minimum *e*_TSh-1_ decreasing to 0.203 and 0.860, respectively. The criterion *e*_TSh-1_ ≥ 0.90 is satisfied only within the parameter intervals of (0.0414, 0.1576) and (0.905, 0.996). Therefore, preference in design should be given to parameter ranges that retain a high TSh-1 modal kinetic energy ratio throughout the manufacturing tolerance range.

The present study focuses on the influence of geometric structures on modal coupling and therefore does not explicitly include electrode effects, electrical boundary conditions, and material damping. For AT-cut quartz resonators, these factors may quantitatively shift the frequency branches of the TSh-1 and parasitic modes and consequently alter the locations of modal anti-crossings. Dissipative effects may also broaden the resonance peaks, reduce modal resolvability, and affect the quality factor. Therefore, geometry-induced low coupling intervals are predicted by the minima of *Γ* and the criterion *e*_TSh-1_ ≥ 0.90. In our recent work [43], different electrode models for thickness-shear AT-cut quartz resonators are systematically investigated. In the future, the present formulation will be extended to an integrated model incorporating electrodes, fully coupled piezoelectric effects, electrical boundary conditions, and structural damping.

## 4. Conclusions

The vibration analysis model for micro AT-cut QMRs with beveled edges was developed based on the first-order Mindlin plate theory for coupled thickness-shear and flexure. The power series solution adopted for the beveled region exhibits stable convergence, and good agreement with the FEM results is obtained for the TSh-1 and adjacent FL branches within the verification range. The results show that the mesa dimensions primarily govern the TSh-1 frequency and energy trapping, whereas excessive thickness discontinuity or an overly long mesa region may enhance interfacial reflection and promote coupling between thickness-shear and flexural modes. The beveled edges have only a limited direct influence on the TSh-1 frequency, but they significantly modify the phase feedback and mode coupling strength of the adjacent FL mode by altering the edge stiffness and wave reflection. The coupling index derived from the Fourier spectrum, as well as the modal kinetic energy ratio, both demonstrate that mesa and beveled-edge parameters have a non-monotonic effect on modal coupling. The appropriate geometric combinations can suppress the FL component mixed into the TSh-1 mode and thereby identify low coupling operating ranges. The fabrication tolerance analysis indicates that the low coupling intervals associated with mesa and bevel lengths are more robust than those associated with mesa height and bevel thickness reduction coefficient. These findings indicate that etched beveled edges should be regarded as a critical geometric factor in the structural design and optimization of micro AT-cut QMRs.

The present formulation will be extended into a fully coupled piezoelectric model by incorporating electrodes, electrical boundary conditions and damping to systematically investigate their combined effects on modal coupling and the quality factor.

## Figures and Tables

**Table 1:** Mesh convergence analysis of finite element model.

Length Ratio *c*/*h*_b_	Free Triangular Mesh Size	Number of Units	TSh-1 Frequency
14.75	finer	515	1.01420
14.75	extra fine	1147	1.01407
14.75	extremely fine	3702	1.01407
18.45	finer	493	1.01396
18.45	extra fine	991	1.01376
18.45	extremely fine	3095	1.01374

**Table 2:** Branch-wise comparison of eigenfrequency errors between theoretical and FEM results.

Frequency Branch	Average Relative Errors%	Maximum Relative Errors%	Length Ratio *c*/*h*_b_
TSh-1	0.04	0.20	19.67
FL-14	0.24	0.37	13.20
FL-16	0.20	0.36	14.95
FL-18	0.27	0.39	16.70
FL-20	0.28	0.40	18.45
FL-22	0.28	0.37	20.00

**Table 3:** Representative FEM calculated eigenfrequencies and mode shapes at selected length-to-thickness ratios.

Length Ratio *c*/*h*_b_	Mode Branch	Ω	Mode Shape	
14.75	FL-16	1.00032	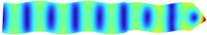	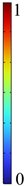
	E-6	1.00764	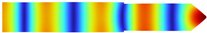	
	TSh-1	1.01407	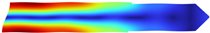	
18.45	FL-20	0.98485	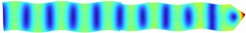	
	E-8	1.00104	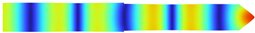	
	TSh-1	1.01376	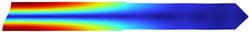	

**Table 4:** Robustness of the predicted low coupling intervals under fabrication tolerances.

Parameter	Nominal Minimum	Fabrication Tolerance	Tolerance Window	Minimum *e*_TSh-1_	Low Coupling Interval	Robustness
*h*_e_/*h*_b_	0.0953	±0.0714	(0.0239, 0.1667)	0.203	(0.0414, 0.1576)	Sensitive
*a*/*h*_b_	7.8261	±0.0357	(7.7904, 7.8618)	0.991	(7.7904, 7.8618)	Robust
	9.0127		(8.9769, 9.0484)	0.993	(8.9769, 9.0484)	
*d*/*h*_b_	0.9596	±0.0714	(0.8882, 1.0310)	0.962	(0.8882, 1.0310)	Robust
	1.2626		(1.1912, 1.3341)	0.942	(1.1912, 1.3341)	
*ξ*	0.9503	±0.0714	(0.8816, 0.9987)	0.860	(0.905, 0.996)	Sensitive

## Data Availability

The original contributions presented in this study are included in the article. Further inquiries can be directed to the corresponding author.

## References

[1] Mao Z.B., Wang J.H., Zhang J.H., Ohgi J.J., Zheng Y.Q., Peng Y.H., Zhao L.Y., Su Q., Huang W.D., Xu B. (2026). Fine-tuned Multimodal Large Language Model for Autonomous State Cognition System of Shape-Recognition 6-bar Tensegrity Integrated with Flexible Sensors. Microsyst. Nanoeng..

[2] Saleh S., Saeidi T., Timmons N., Razzaz F. (2024). A Comprehensive Review ofRecent Methods for Compactness and Performance Enhancement in 5G and 6G Wearable Antennas. Alex. Eng. J..

[3] Devi D.H., Duraisamy K., Armghan A., Alsharari M., Aliqab K., Sorathiya V., Das S., Rashid N. (2023). 5G Technology in Healthcare and Wearable Devices: A Review. Sensors.

[4] Xu F.M., Yin C.Y., Chen J.Y., Dou G.B., Sun L.T. (2025). Design Strategy and Micromachining Technology of AT-cut High-frequency Quartz Resonators: A review. Mater. Sci. Semicond. Process..

[5] Nair M.P., Teo A.J., Li K.H.H. (2021). Acoustic Biosensors and Microfluidic Devices in the Decennium: Principles and Applications. Micromachines.

[6] Arnau A. (2008). A Review of Interface Electronic Systems for AT-cut Quartz Crystal Microbalance Applications in Liquids. Sensors.

[7] Ma L.X., Zhou Q., Yi L.J., Wang J. (2026). Unified Model for Force-frequency Coefficient of Different Shape Quartz. Int. J. Mech. Sci..

[8] Yang G., Huang X.H., Tan K., Chen Q., Pan W. (2023). Study of Force-frequency Characteristics in AT-cut Strip Quartz Crystal Resonators with Different Rotation Angles. Sensors.

[9] Shen J.Q., Chen C.Y., Wu C.Y., Cheng J.G., Chao M.C., Zhou Q., Lu C.D. (2024). The Force–Frequency Characteristics of Quartz Wafers under a Cantilever Beam Structure. Sensors.

[10] Sun Z.B., Wang Z., Li Z., Guo Y., Huang B. (2024). High-frequency Vibration of Beveled Crystal Plates by Using Subregional Geometric Fitting Method. Sci. Rep..

[11] Li B., Li C., Zhao Y.L., Han C., Zhang Q.W. (2020). Deep Reactive Ion Etching of Z-Cut Alpha Quartz for MEMS Resonant Devices Fabrication. Micromachines.

[12] Zhang J.L., Liao S., Chen C., Yang X.T., Lin S.A., Tan F., Li B., Wang W.W., Zhong Z.X., Zeng G.G. (2021). Research on Trimming Frequency-Increasing Technology for Quartz Crystal Resonator Using Laser Etching. Micromachines.

[13] Wan Y., Luan X.H., Zhou L.Z., Wu F.S. (2022). Wet Etching of Quartz Using a Solution Based on Organic Solvents and Anhydrous Hydrofluoric Acid. Materials.

[14] Liang J.X., Huang J., Zhang T., Zhang J., Li X.F., Ueda T. (2013). An Experimental Study on Fabricating an Inverted Mesa-Type Quartz Crystal Resonator Using a Cheap Wet Etching Process. Sensors.

[15] Takashi A., Ngoc H.V., Masayoshi E. (2006). Inverted Mesa-type Quartz Crystal Resonators Fabricated by Deep-reactive Ion Etching. IEEE Trans. Ultrason. Ferroelectr. Freq. Control.

[16] Yan X., Jin Z., Gosálvez M.A., Hui Z., Yuan L., Hua Z.S. (2018). The Maximum Positive Curvature Recognition Method to Determine Etch Profiles in Wet Etching of Quartz on AT and BT Cuts. J. Microelectromech. Syst..

[17] Yan X., Gosálvez M.A., Hui Z., Yuan L., Li Q.X. (2017). Transient and Stable Profiles During Anisotropic Wet Etching of Quartz. J. Microelectromech. Syst..

[18] Qiang X.L., Chun X.J., Xu W.H., Tao D.P., Zhong W.X. (2012). Forecast of Structure Sidewall Profiles for z-cut Quartz after Anisotropic Wet Etching. Opt. Precis. Eng..

[19] Wen C.K., Yin C.Y., Xu F.M., Dou G.B. (2026). High Aspect Ratio Through-Wafer Etching of AT-Cut Quartz Using Double-Sided Offset Apertures. Sens. Actuators A Phys..

[20] Shen F., Lu P., O’Shea S.J., Lee K.H. (2003). Frequency Coupling and Energy Trapping in Mesa-Shaped Multichannel Quartz Crystal Microbalances. Sens. Actuators A Phys..

[21] Lu F., Lee H.P., Lim S.P. (2005). Energy-trapping Analysis for the Bi-Stepped Mesa Quartz Crystal Microbalance Using the Finite Element Method. Smart Mater. Struct..

[22] He H.J., Liu J.X., Yang J.S. (2011). Thickness-Shear and Thickness-Twist Vibrations of an AT-Cut Quartz Mesa Resonator. IEEE Trans. Ultrason. Ferroelectr. Freq. Control.

[23] He H.J., Nie G.Q., Liu J.X., Yang J.H. (2012). Energy Trapping of Thickness-Shear and Thickness-Twist Modes in a Partially Electroded AT-Cut Quartz Resonator. Acta Mech. Solida Sin..

[24] Zhu F., Wang B., Dai X.Y., Qian Z.H., Kuznetsova I., Kolesov V., Huang B. (2019). Vibration Optimization of an Infinite Circular AT-Cut Quartz Resonator with Ring Electrodes. Appl. Math. Model..

[25] Ma T.F., Wang J., Du J.K., Yang J.S. (2015). Resonances and Energy Trapping in AT-Cut Quartz Resonators Operating with Fast Shear Modes Driven by Lateral Electric Fields Produced by Surface Electrodes. Ultrasonics.

[26] Li P., Jin F., Yang J.S. (2012). Thickness-Shear Vibration of an AT-Cut Quartz Resonator With a Hyperbolic Contour. IEEE Trans. Ultrason. Ferroelectr. Freq. Control.

[27] Peng L., Feng J. (2018). The Investigation of Trapped Thickness Shear Modes in a Contoured AT-Cut Quartz Plate Using the Power Series Expansion Technique. J. Phys. D. Appl. Phys..

[28] Abe T., Kishi H. (2010). A Gaussian-shaped AT-cut Quartz Crystal Resonator. Sens. Actuators A Phys..

[29] Wenjun W., Rongxing W., Ji W., Jianke D., Jiashi Y. (2013). Thickness-Shear Modes of an Elliptical, Contoured AT-Cut Quartz Resonator. IEEE Trans. Ultrason. Ferroelectr. Freq. Control.

[30] Lee P.C.Y., Wang J. (1996). Thickness-Shear and Flexural Vibrations of Contoured Crystal Strip Resonators. J. Appl. Phys..

[31] Wang J., Lee P.C.Y., Bailey D.H. (1999). Thickness-Shear and Flexural Vibrations of Linearly Contoured Crystal Strips with Multiprecision Computation. Comput. Struct..

[32] Li N.A., Wang B., Qian Z.h. (2018). Coupling Vibration Analysis of Trapped-Energy Rectangular Quartz Resonators by Variational Formulation of Mindlin’s Theory. Sensors.

[33] Li M.J., Li N.A., Li P., Liu D.Z., Kuznetsova I.E., Qian Z.H., Ma T.F. (2023). The Multi-field Coupled Vibration Analysis of AT-Cut Quartz Crystal Resonators with Parallelism Error. Acta Mech. Solida Sin..

[34] Li M.J., Li P., Li N.A., Liu D.Z., Kuznetsova I.E., Qian Z.H. (2023). Surface Roughness Effects on the Vibration Characteristics of AT-Cut Quartz Crystal Plate. Sensors.

[35] Lee P.C.Y., Wang J. (1994). Vibrations of AT-cut Quartz Strips of Narrow Width and Finite Length. J. Appl. Phys..

[36] Chen G.J., Wu R.X., Wang J., Du J.K., Yang J.S. (2012). Five-mode Frequency Spectra of x 3-dependent Modes in AT-cut Quartz Resonators. IEEE Trans. Ultrason. Ferroelectr. Freq. Control.

[37] Mindlin R.D. (2006). An Introduction to the Mathematical Theory of Vibrations of Elastic Plates.

[38] Hu Y.T., Hu H.P., Yang J.S. (2006). A Low Frequency Piezoelectric Power Harvester Using a Spiral-shaped Bimorph. Sci. China Phys. Mech. Astron..

[39] Zhao Z., Zhu X.Z., Chen W.Q. (2026). Temperature-driven Vibration Transitions in Mode-coupled Piezoelectric Resonators. Int. J. Mech. Sci..

[40] Jiang S., Hu H.P., Laude V. (2018). Low-frequency band gap in cross-like holey phononic crystal strip. J. Phys. D. Appl. Phys..

[41] Jiang S., Hu H.P., Laude V. (2018). Ultra-wide band gap in two-dimensional phononic crystal with combined convex and concave holes. Phys. Status Solidi–Rapid Res. Lett..

[42] Goka S., Tamura T., Sekimoto H., Watanabe Y., Sato T., Sato K. (2004). Mode Decoupling Effect of Multistepped Bi-Mesa AT-Cut Quartz Resonators. Jpn. J. Appl. Phys..

[43] Chen H., Fu X., Yang W., Zhan C., Xiong F., Wang X., Hu H., Hu Y. (2026). Comparative Performance of Electrode Models for Thickness-Shear AT-Cut Quartz Resonators. Micromachines.

